# The Case Against Human Rights Penality

**DOI:** 10.1093/ojls/gqae013

**Published:** 2024-04-30

**Authors:** Natasa Mavronicola

**Affiliations:** Professor of Human Rights Law, Birmingham Law School, University of Birmingham, Birmingham, United Kingdom

**Keywords:** human rights, penality, positive obligations

## Abstract

This article seeks to make the human rights case against human rights penality—that is, against the reliance upon and *foregrounding* of penal mechanisms in the protection of (certain) human rights. The rationale for the alliance between human rights and state penality has at least three central dimensions: effectiveness, accountability and equal protection. In particular, the mobilisation of criminal law (enforcement) and punishment is often treated as the most effective means of preventing and/or redressing human rights violations. Moreover, the criminal process and sanction are often understood as the pinnacle of accountability for serious human rights violations. Finally, the egalitarian rationale for human rights penality views it as redistributing protection to under-protected persons. While remaining committed to human rights, I unpack (some of) the ways in which human rights penality ultimately fails to uphold and even undermines the principles that it has been promoted as fulfilling within the human rights frame.

## 1. Introduction

Human rights law is associated with the protection of the person from abuse and arbitrariness in the exercise of the state’s penal power. Human rights actors frequently condemn the many instances of individual and systemic brutality inflicted by the coercive and carceral institutions of the state, from police violence—often racist police violence—to the degradation that is rife within prisons and other carceral settings around the world. Increasingly, such actors are also acknowledging the institutional prejudice, systemic racism and wider structural injustice in which these abuses are embedded, as demonstrated in a statement issued by several United Nations mandate-holders in the aftermath of the killing of George Floyd in the United States.^1^

At the same time, human rights actors not only tolerate the existence of the police and the prison;^2^ they also *compel* it. Human rights law is replete with positive obligations to criminalise, police, criminally investigate, prosecute and punish human rights violations.^3^ This phenomenon, whereby human rights law mobilises, relies upon and foregrounds states’ (most) coercive and carceral apparatus, may be referred to as ‘human rights penality’,^4^ or ‘coercive human rights’.^5^

As I argue in this article, the reasoning that underpins human rights penality has three central pillars. These are: effectiveness, accountability, and equal protection. The first rationale relates to penality’s (perceived) potency: this views the penal apparatus as the most effective mechanism for preventing, protecting people from, and/or redressing human rights violations. In addition, the criminal process and the penal sanction tend to be understood as the pinnacle of accountability for serious human rights violations. Finally, the egalitarian dimension of human rights penality sees duties to prosecute and punish as being oriented at redistributing meaningful protection to under-protected persons.^6^

This article presents the case against human rights penality—that is, against the reliance upon and *foregrounding* of penal mechanisms in the protection of (certain) human rights—from the perspective of a constructively critical supporter of human rights. Taking the rationales identified above seriously, it unpacks how human rights penality fails to uphold or even undermines them. In other words, it contemplates the ways in which human rights penality is bad for human rights. A growing body of work, including my own, critiques the coercive orientation of human rights norms from various angles, often as part of a more wide-ranging critical stance towards human rights law and international criminal law.^7^ This article makes the *human rights* case against human rights penality: building on but also departing from this growing body of work, it identifies the central rationales of human rights penality and offers a novel taxonomisation of its limitations and harms; and it does so *from within* human rights. In other words, the critique of human rights penality offered in this article begins from a commitment to the fundamental tenets of human rights law, and argues that human rights penality does not serve them.

The focus of this article is international human rights law—as distinct from international criminal law or other domains of international law—and its ‘sword function’^8^ in mobilising, rather than shielding from, the state’s penal apparatus. In my analysis and critique of human rights penality, I rely primarily on the pronouncements of the European Court of Human Rights (ECtHR), the Inter-American Commission of Human Rights (IACommHR), the Inter-American Court of Human Rights (IACtHR) and the United Nations Human Rights Committee. My references to relevant international legal instruments and jurisprudence are meant to be illustrative rather than comprehensive. The article proceeds as follows. Section 2 briefly introduces the phenomenon of human rights penality. Section 3 elaborates the central rationales underpinning the development of and primacy given to obligations to mobilise the criminal law as a (or the) means of protecting from, and offering redress for, human rights violations. It argues that human rights penality is chiefly grounded in its perceived connection to three fundamental aims of human rights law and doctrine: effectiveness, accountability and equal protection.

Against the background of these widely articulated rationales for human rights penality, section 4 identifies five ways in which human rights penality is bad for human rights. The five problematic implications of human rights penality identified are: the dilution of human rights standards; the distortion of principles closely aligned to foundational commitments within human rights; the diversion of human rights law and doctrine from critical dimensions of human rights protection; the hollowing out of visions of justice within the human rights frame; and the delegitimisation of progressive agendas. The article concludes with an appeal to those committed to human rights to acknowledge the significant limitations, risks and (opportunity) costs of human rights penality, and to reimagine human rights law and doctrine in more meaningfully protective and just terms.

## 2. Penality in Human Rights Law and Doctrine

Although human rights have traditionally been cast as a ‘shield’ against the state’s coercive and carceral power, they have increasingly cemented their ‘sword’ function, by demanding the mobilisation of criminal law (enforcement) and punishment.^9^ Today, penality—understood as the mobilisation of the criminal law, criminal process and criminal punishment, and discursive appeals to the perceived protective potency of such mechanisms^10^—pervades human rights law and doctrine.

Penality has been foregrounded in a range of legal instruments.^11^ Widely ratified treaties, such as the United Nations Convention Against Torture^12^ and the Convention for the Protection of All Persons from Enforced Disappearances,^13^ not only established duties to criminalise, prosecute and punish various human rights violations, but also made them their centrepiece.

Penality has also taken centre stage in pronouncements of various judicial and oversight bodies within the human rights edifice, becoming the defining feature of positive obligations attached to human rights whose violation is deemed particularly serious, such as the right to life or the right not to be subjected to torture or other cruel, inhuman or degrading treatment or punishment.^14^ Across the human rights edifice, key human rights norms such as these have been authoritatively interpreted as giving rise to positive obligations on the state to criminalise, police, prosecute and punish their violation.^15^ The IACtHR and IACommHR have been at the forefront of these developments,^16^ followed closely by the ECtHR and the United Nations Human Rights Committee.^17^

The development of human rights penality within judicial pronouncements is illustrated in a recently published volume entitled *Coercive Human Rights*, which contains a multifaceted analysis and critical examination of duties to criminalise, police, prosecute and punish under the European Convention on Human Rights (ECHR).^18^ As the book illustrates, states’ positive obligations under a range of human rights norms have tended to be specified by the ECtHR as requiring the mobilisation of criminal law (enforcement) as the central means of protection from, and redress for, human rights violations. This is the case across all ‘categories’ of positive obligation. In ECtHR jurisprudence on certain key rights, the general protective duty of the state to establish an adequately protective legal framework and ensure it is properly implemented is largely understood as necessitating a criminal law framework and robust criminal law enforcement.^19^ States’ operational protective duties, which arise in situations where the authorities know or ought to know of a real and immediate risk of serious harm to particular persons,^20^ are chiefly understood as necessitating policing operations.^21^ Finally, the procedural duty to investigate where there is an allegation or credible suspicion of a serious human rights violation tends to be understood as demanding an investigation capable of leading to ‘the identification and *punishment* of those responsible’.^22^ This generally translates into a requirement to undertake a criminal investigation governed by the criminal process.

Similar approaches transpire in the pronouncements of other human rights bodies, whereby rights whose violation is deemed ‘serious’ are understood as giving rise to duties to mobilise state penality.^23^ The IACtHR considers that ‘criminalization is an appropriate way to protect certain rights’,^24^ and has viewed the obligation to prevent certain human rights violations as requiring that ‘any violations are considered and treated as illegal acts, which, as such, may lead to the punishment of those responsible’.^25^ In turn, the Human Rights Committee has increasingly emphasised duties to criminalise, and to conduct criminal investigations into, violations of rights such as the right to life and the right not to be subjected to torture or ill-treatment.^26^

Human rights penality has developed against the backdrop of a broader anti-‘impunity’ agenda in the international legal arena.^27^ The anti-impunity agenda places emphasis on holding perpetrators of serious violations of international law criminally accountable, helping shape a legal and institutional framework founded on the understanding that grave wrongdoing must be punished.^28^ From the Nuremberg Trials to the development of the *ad hoc* international criminal tribunals and the International Criminal Court,^29^ the pursuit of individual criminal accountability with respect to deeply egregious wrongs against persons, or against humanity itself,^30^ has become the ‘justice norm’ around which substantial consensus has built.^31^

## 3. The Rationales for Human Rights Penality

Why has penality been promoted and even foregrounded in human rights law? The answer to this question lies in the widespread conviction that it advances some of the fundamental aims of human rights. It is, I would argue, principally tied to three central rationales: effectiveness, accountability and equal protection. In this section, I outline how human rights penality is understood to advance these ideals.

### A. Effectiveness

In developing human rights penality, bodies such as the IACtHR, IACommHR and ECtHR have repeatedly lauded the ‘effectiveness’ of criminal law tools in securing human rights guarantees for (potential) victims of human rights violations. As Anja Seibert-Fohr has put it, ‘According to the [Inter-American] Commission, adjudication and punishment are the single most effective means to enforce human rights’.^32^ For the IACtHR, the efficient mobilisation of the criminal process is the way to ensure ‘effective judicial protection’^33^ of key rights. Indeed, even as it has acknowledged that the effectiveness of domestic legal measures is attested by *conformity* with the relevant norms as much as it is by their punitive implementation,^34^ the IACtHR has ultimately adopted a stance that equates *guaranteeing* certain rights with adequately punishing their violation. In *Garrido and Baigorria v Argentina*, a case concerning enforced disappearances, it reasoned:

Argentine laws that guarantee the right to life have been violated in the instant case. Therefore, to ensure their effectiveness, Argentina must *apply* the provisions established for violations of those laws; in other words, it must impose the corresponding *sanctions*. These are the measures provided for in the American Convention and that the State must take to ensure the effectiveness of the rights and duties guaranteed under the Convention.^35^

At the ECtHR, the advance of human rights penality has been closely tied to the principle that ‘the Convention is intended to guarantee rights that are not theoretical or illusory, but practical and effective’,^36^ a mantra with which the ECtHR has repeatedly prefaced its delineation of coercive duties.^37^ Paul Lemmens and Marie Courtoy argue that the ECtHR views it as necessary ‘to have in place legislation with a deterrent effect, followed by concrete measures showing that the authorities are not prepared to allow the offences to go unpunished, in order to prevent the commission of particularly wrongful acts’.^38^ On this account, the criminal law and criminal sanction ensure the effective protection of human rights by *deterring* human rights violations.^39^ Indeed, the position often adopted by the ECtHR is that an absence of a criminal law mechanism or criminal law response entails *ineffectiveness* in protecting certain human rights. In its first ‘coercive human rights’ judgment in 1985, *X and Y v Netherlands*, the ECtHR underlined that the case concerned ‘fundamental values and essential aspects of private life’, whose protection required ‘effective deterrence’, which could be ‘achieved *only* by criminal-law provisions’.^40^ In the domestic violence case of *Volodina v Russia*, the ECtHR posited that ‘the State authorities have a responsibility to take *protective* measures in the form of effective *deterrence* against serious breaches of an individual’s personal integrity by a member of her family or by a partner’.^41^ This stance is also reflected in the Human Rights Committee’s clarification that anything *but* a criminal law response would be inadequate in addressing suspected violations of the right to life.^42^

Within these strands of reasoning shaping human rights penality, ‘impunity’, understood in narrow terms as the ‘inadequate’ mobilisation of criminal punishment, becomes synonymous with the ineffective protection of human rights. Mobilising penality in dealing with human rights violations therefore becomes ‘unquestionable common sense’ for anyone eager to see human rights effectively protected.^43^

### B. Accountability

Accountability may be broadly understood to mean rendering account in respect of a particular wrong, and making relevant amends.^44^ While this understanding of accountability can encompass a variety of actors and a wide-ranging understanding of the harm(s) and wrong(s) at issue, the idea of accountability for what are deemed ‘serious’^45^ human rights violations has, with the growth of human rights penality, been chiefly understood in individualised terms as necessitating the establishment and deployment of an ‘apparatus aimed at holding perpetrators accountable’.^46^ Accountability for such violations has thereby often (though not always) been used interchangeably with criminal redress, and closely associated with the ascent of international criminal law and international criminal justice.^47^

The ECtHR has repeatedly underlined that the investigative duty’s purpose is the ‘identification and punishment of those responsible’ for the human rights violation at issue.^48^ For the ECtHR, this particular form of accountability—that is, holding individual perpetrators criminally accountable for the wrongdoing at issue—is seen as securing not only appropriate redress, but also effective protection through deterrence. The Court’s reasoning in *Öneryildiz v Turkey*, a case concerning an explosion at a municipal rubbish tip that caused multiple deaths, is instructive in this respect. The Court found that, in undertaking limited proceedings which resulted in ‘derisory’ and suspended fines for relevant officials,^49^ the Turkish criminal justice authorities had not ‘secured the full accountability of State officials … and … the effective implementation of provisions of domestic law guaranteeing respect for the right to life, in particular the deterrent function of the criminal law’.^50^

The notion that accountability is the opposite of impunity, and that its fulfilment requires the pursuit of criminal investigation, prosecution and (adequate) punishment, is also well embedded within the pronouncements of the IACommHR and IACtHR.^51^ For the IACtHR, impunity means ‘the total lack of investigation, prosecution, capture, trial and conviction of those responsible for violations of the rights protected by the American Convention’, and the resultant fostering of ‘chronic recidivism of human rights violations, and total defenselessness of victims and their relatives’.^52^

According to the Human Rights Committee, too, the pursuit of accountability goes hand in hand with ‘preventing impunity’.^53^ The close connection between accountability and the prevention of impunity has been underscored in General Comment 36 (on the right to life) alongside the Committee’s emphasis on the imperative of seeking criminal redress for violations of the right to life,^54^ thereby demonstrating the tendency to understand accountability as individual criminal accountability.

The pursuit of accountability through penality is often tied to an even loftier ideal: *justice*. The prosecution and punishment of perpetrators of serious human rights violations is seen as a way—or often *the* way—of achieving justice for what victims/survivors or their loved ones have endured.^55^ On this basis, Kathryn Sikkink has traced what she terms a ‘justice cascade’: a legitimisation of ‘the norms of individual criminal accountability for human rights violations and an increase in actions (such as trials) on behalf of those norms’.^56^ Conversely, a lack of prosecution and punishment in response to serious human rights violations—notably through amnesties—is often deemed to amount to injustice.^57^ Human rights penality therefore translates accountability into individual criminal accountability, that is, the identification and punishment of those responsible, and can tend towards confining the dominant understanding of justice for serious human rights violations to retributive justice within the frame of the criminal process.^58^

### C. Equal Protection

In *Coercive Human Rights*, a number of authors praise the ECtHR’s coercive duties jurisprudence with particular reference to domains such as gender-based violence,^59^ the abuse of children,^60^ and hate speech against marginalised persons.^61^ Stephanos Stavros writes approvingly of the promise held by the ECtHR’s growing recognition of hate speech as a phenomenon to which a criminal response is not just permitted but *required* under human rights law. He sees the relevant obligations as sending ‘a powerful message concerning the weight attached by the Court to the need to protect the most vulnerable members of society’.^62^ Corina Heri comments positively on the link between the Court’s elaboration of coercive duties and its recognition of the vulnerability of the victims of wrongs to which it demands a coercive response. She argues that ‘Vulnerability, in this context, serves as a lens that focuses attention on those victims who are least likely to be treated respectfully, believed, heard or granted justice’.^63^ These accounts provide an important insight into another key rationale of human rights penality: that of equal protection. They present key advancements in human rights penality as upholding the rights and dignity of the marginalised, the vulnerable, the under-protected. Here, the significance of human rights penality is seen to lie not only in the concrete redress or effective protection it purportedly offers to those who have been under-protected, but also in the *expressive* function of attaching criminal sanctions to such wrongdoing. This emerges explicitly in Alina Balta’s contribution in the same volume, where she argues that retributive responses to grave human rights violations can play a significant part in ‘the rebalancing of the worth and social standing of victims’ within the affected community.^64^

Indeed, impunity, as the target of human rights penality, is seen to embody not only ineffectiveness and injustice, but also inequality. Impunity is viewed as reflecting a certain imbalance of power as well as an imbalance of *regard* for those left ‘defenceless’, as the IACtHR puts it.^65^ Condemning impunity, therefore, means condemning a reality in which all too many marginalised persons are over-policed and under-protected,^66^ while those who wield power and privilege often receive little to no sanction for their wrongdoing, particularly when their conduct harms those on the margins of society’s regard. Human rights penality, then, seeks to ‘turn the canons of the punitive machine against the powerful’,^67^ to better address so-far under-recognised and under-addressed wrongs (such as gender-based violence) and better protect under-protected persons (such as racialised minorities). Hadar Aviram labels this egalitarian drive to turn the punitive machine against the powerful ‘progressive punitivism’.^68^ The egalitarian aim of protecting the heretofore persecuted, and of piercing the armour shielding the privileged from the consequences of their abuses, is particularly prominent in the aftermath of widespread violence and injustice. Reclaiming or restoring victims’ equal moral status in society is, therefore, a dominant theme in the pursuit of penal accountability in transitional contexts.^69^

## 4. Reasons to Rethink Human Rights Penality

The above account distils how human rights penality is rationalised as securing practical and effective protection of rights, holding perpetrators properly accountable, and confronting a status quo that shields the powerful and abandons the powerless. However, as I argue in the rest of this article, human rights penality is at best a blunt instrument for achieving the laudable aims with which it has been associated. Besides penality’s inadequacy in securing effective and equal protection for all (potential) victims of human rights violations and attaining full accountability in the aftermath of such abuses, its growing hold on human rights carries significant dangers and ongoing harms to human rights. Below, I outline five reasons why human rights penality is bad for human rights.

### A. The Problem of Dilution

The notion that human rights penality *enhances* the potency of human rights norms is undermined by the fact that it can operate, and has operated, to dilute stringent human rights standards for holding states responsible for human rights violations. The growing centrality of criminal law and criminal punishment to human rights norms has created pressures towards ‘alignment’ between human rights and the criminal law. Alignment here means treating human rights violations as coextensive with criminal wrongs, or vice versa.^70^ Such alignment can stem from attempts to ensure that the domestic legal protection mandated by human rights ‘mirrors’ the international or regional standard applicable. Yet, instead of enhancing the potency of human rights norms, alignment—that is, treating the substantive scope of (certain) human rights as coterminous with criminal law norms—can have the implication of weakening the demanding standards through which state authorities are held to account.

The problem is as follows. If certain human rights violations, such as inhuman or degrading treatment, or a violation of the right to life, are deemed to warrant a criminal-law response, then the corresponding rights may come to be seen as proscribing *only criminal wrongs*. This entails a narrowing of what we currently understand to be the wrongs captured by human rights, such as the right to life or the right not to be subjected to torture or inhuman or degrading treatment.^71^ Human rights violations—even what are often referred to as *serious* human rights violations—do not always involve criminally culpable behaviour. For example, the right not to be subjected to torture or to other cruel, inhuman or degrading treatment or punishment concers not only conduct that bears the hallmarks of intent and purpose that are typical elements of torture,^72^ but also circumstances that do not involve criminally culpable behaviour.^73^ This can include ill-treatment that is inflicted via legal regime, such as life imprisonment without parole^74^ or restrictions on reproductive autonomy,^75^ which may not entail individual criminal liability. The right also captures situations where the responsibility or culpability of relevant actors is diffuse or otherwise not criminal in character—this may be the case in many instances of cumulatively degrading prison conditions, for example.^76^ These situations are rightly captured by the right—they represent ways in which persons are profoundly wronged, often in carceral institutional settings, even if there is no criminally culpable perpetrator involved. The risk posed by alignment is that relevant human rights bodies may feel compelled to read such rights more narrowly or more 'leniently' so as to capture only acts and omissions that are culpable enough to warrant criminal sanction.

This prospect is not merely speculative. Such dilution is already materialising in ECtHR doctrine.^77^ In particular, in case law on the right to life under Article 2 ECHR, the obligation not to use lethal force except in circumstances where such force is absolutely necessary has been read down so as to catch only the most incontrovertibly criminal conduct.

Article 2(2) ECHR provides that the deprivation of life is not to be regarded as inflicted in contravention of the right to life when it results from the use of force which is *no more than absolutely necessary* in defence of any person from unlawful violence, or other aims (such as effecting an arrest or quelling a riot) which are ultimately subsumed under the defence of self or others criterion.^78^ The ECtHR has repeatedly underlined that

the use of the term ‘absolutely necessary’ in Article 2(2) indicates that a stricter and more compelling test of necessity must be employed from that normally applicable when determining whether State action is ‘necessary in a democratic society’ under paragraph 2 of Articles 8 to 11 of the Convention.^79^

This suggests a strict assessment of the *objective* necessity of the use of potentially lethal force, given that the proportionality test applicable to qualified rights already encompasses an objective criterion of necessity.

However, ‘absolute necessity’ has come to be read as an entirely subjective standard, with the criterion of ‘honest belief’ superimposed upon it. Specifically, the ECtHR has now established that the use of potentially lethal force ‘may be justified under this provision where it is based on an honest belief which is perceived, for good reasons, to be valid at the time but which subsequently turns out to be mistaken’.^80^ While the ‘honest belief’ criterion appeared initially to be qualified by an objective prerequisite—that the honest belief must be held ‘for good reasons’—the Grand Chamber of the ECtHR has now explicitly dispelled this notion. In *Da Silva v UK*, the Court confirmed that it considers that ‘the existence of “good reasons” should be determined subjectively’.^81^ Importantly, the dilution of the seemingly objective criterion of ‘good reasons’ in *Da Silva* was shaped by the Court’s determination that the right to life under Article 2 ECHR aligned with the English criminal law on homicide. This finding was made in response to arguments from the applicant and third-party interveners that English criminal law did *not* reflect the more stringent Article 2 absolute necessity standard on the use of force.^82^

The message this sends on the compatibility of lethal force with Article 2 ECHR is that such compatibility hinges on whether the person employing such force ‘had an honest and genuine belief that the use of force was necessary’.^83^ Besides the significant dilution of the obligation not to take life that it brings about, this approach also appears to accommodate all subjectively held reasons for holding such a ‘genuine’ belief, not least harmful racial stereotypes. The potential for profound injustice being deemed human rights-compatible is evident here, and clearly undercuts the rationales of effective and equal protection underpinning human rights penality, as well as its association with justice.

Other rights also stand to be narrowed to exclude conduct that lacks criminal(-like) culpability, thereby undermining significant advances made through the methodical development of so-called ‘expansive’^84^ readings of human rights. There is cause for concern regarding such a redirection in approaches to the right not to be subjected to torture or inhuman or degrading treatment. In *Nicolae Virgiliu Tănase v Romania*, the ECtHR’s Grand Chamber reasoned that the duty to investigate under Article 3 ECHR was inapplicable to a car accident by stressing that Article 3 ill-treatment must be ‘the consequence of an intentional act’.^85^ While the ECtHR was arguably correct to find that Article 3 does not apply by default to car accidents, the Court’s reasoning appears to incorporate a requirement of a *mens rea* of intent—a frequent marker of criminally culpable conduct—into what ‘counts’ as Article 3 ill-treatment. In his vigorous criticism of the majority’s reasoning in this case, Judge Kūris pointed specifically to prison conditions—which are frequently the subject of adverse Article 3 findings by the ECtHR—as often involving ill-treatment that is not necessarily ‘the consequence of an intentional act’.^86^ Similarly troubling reasoning, focusing on the absence of an intention to ill-treat, was used more recently to determine that the forced sterilisation of a woman during childbirth did not violate Article 3 ECHR.^87^ In *YP v Russia*, the majority underlined that ‘the doctors had not acted in bad faith, let alone with an intent of ill-treating or degrading her’, and concluded that, ‘albeit clearly disrespectful of the applicant’s autonomy’, the decision to sterilise was therefore not contrary to Article 3 ECHR.^88^ In concurring with this finding, Judge Elósegui repeatedly associated Article 3 violations with the pursuit of criminal liability. She insisted that ‘the element of intent is very important to the application of Article 3’^89^ and that it is ‘doubtful to apply that Article to doctors who have acted in good faith, even if they were wrong’.^90^ According to her, ‘if Article 3 is applied to all these situations (even in the absence of any intent) the door would be open to all kinds of accusations against health personnel by criminal avenues’.^91^

If alignment operates to narrow the scope of circumstances in which the state is found to be in violation of Article 3 ECHR, it is capable of eroding much of the right’s protection in a range of contexts where inhumanity or degradation are frequent or indeed pervasive, whether inflicted unintentionally, through a legal regime or as a result of structural, systemic, diffuse or even individual failings, errors or wrongdoing that simply do not make up an individual criminal wrong. These would include the imposition of a *de facto* irreducible sentence of life imprisonment,^92^ degrading prison conditions in inadequately resourced prisons,^93^ or substantively flawed asylum judgments resulting in the removal of persons to places where they face a real risk of torture or inhuman or degrading treatment or punishment.^94^ Moreover, a tendency towards alignment, with the concomitant emphasis on criminal markers of culpability such as intent to cause harm, can lend credence to arguments seeking to stymie the right or to suppress some of its least ‘popular’ elements, notably those outlined above.^95^ It should be a source of profound concern if efforts to roll back human rights protections stand to be bolstered by human rights bodies’ increasing tendency to align key human rights norms with the criminal law. It should also lead to profound questioning of the effectiveness rationale driving human rights penality.

It is not only the substantive content of human rights that is threatened by a tendency towards alignment between human rights and the criminal law. Also at risk are critically important evidentiary standards through which human rights cases are adjudicated with due acknowledgement of the power and information imbalances at play. Returning to the ECHR, the ECtHR has tended to refer to an evidentiary standard of ‘beyond reasonable doubt’ as being needed to establish violations of fundamental rights, such as the right to life and the right against torture.^96^ ‘Beyond reasonable doubt’ is suggestive of a criminal standard orientated at protecting the accused wrongdoer.^97^ Yet the Court has long tended to avoid applying such a demanding test in practice, being much more open to inferences and presumptions of wrongdoing, conscious of the asymmetry in power and knowledge between individual persons and state authorities. For example, the Court applies a presumption that injuries occurring in state custody were the result of proscribed ill-treatment for which state agents are responsible.^98^

Similar flexibility and sensitivity to context are displayed by other human rights bodies in determining alleged human rights violations.^99^ Indeed, from as early as 1989, the IACtHR defended the principle that a flexible standard of proof must be employed in proceedings before the Court by underlining that:

The international protection of human rights should not be confused with criminal justice. States do not appear before the Court as defendants in a criminal action. The objective of international human rights law is not to punish those individuals who are guilty of violations, but rather to protect the victims and to provide for the reparation of damages resulting from the acts of the states responsible … In contrast to domestic criminal law, in proceedings to determine human rights violations the State cannot rely on the defense that the complainant has failed to present evidence when it cannot be obtained without the State’s cooperation.^100^

A tendency towards alignment with the criminal law threatens the much-needed flexibility exemplified in the above excerpt, carrying the risk of a retreat from the open and context-sensitive ways in which human rights bodies distribute the burden of proof, appraise relevant evidence and determine violations, thereby making it harder for those who have suffered human rights abuses to prove the relevant violations.

Again, this concern is not merely speculative. Recent ECtHR case law on the right to life suggests that the ECtHR has been unduly reticent in finding the state to have violated the right to life, employing the criterion of ‘beyond reasonable doubt’ to shield state authorities from reasonable inference of violation.^101^ The Court has similarly employed the ‘beyond reasonable doubt’ standard to refuse, in several cases, to infer a racial discrimination element in findings of violation of Article 2 or 3 ECHR.^102^ Such a rigid approach to the standard of proof accords the state a robust presumption of innocence and a degree of protection from condemnation that should be reserved for individual persons facing criminal punishment. All of this stands at odds with the notion that human rights penality strengthens human rights norms.

### B. The Problem of Distortion

The distorting implications of alignment between human rights and the criminal law are evident. Such alignment can mean that those adjudicating human rights violations treat the apparatus of the state with the leniency that is to be afforded to imperfect human beings facing potentially carceral punishment under criminal law standards, rather than the stringency warranted by the power that this state apparatus wields over us.

The distortion caused by human rights penality goes further than that, however. As Nina Peršak has highlighted,^103^ demanding the mobilisation of the criminal law and its enforcement apparatus as the foremost means of effectively protecting (certain) human rights threatens to subvert the principle of ‘criminal law as last resort’.^104^ This principle, which is also known as the principle of *ultima ratio*, is often appealed to as a constraint on the criminalisation of human conduct. It encapsulates the idea that the criminal law should only be employed where other, less liberty-restricting and dignity-endangering means have been shown to be inadequate, and where resort to the criminal law has been affirmatively shown to be more effective and desirable in dealing with the issue. This aversion to penality is premised on recognition of the ways in which criminalisation can impinge upon or threaten a range of fundamental rights, including but not limited to the right to liberty. Upholding the principle of *ultima ratio* would appear to be in line with the very underpinnings of human rights, at whose heart is ‘respect for human dignity and human freedom’.^105^

In spite of this, human rights penality has seen human rights bodies often treat the criminal law and its apparatus as the *first*, rather than the last, resort. Such an approach may be observed in the readiness with which the IACtHR has demanded punishment for violations of human rights,^106^ and in the ECtHR’s often immediate recourse to the criminal law and its enforcement as the main tools for preventing and redressing certain human rights abuses.^107^ Similarly, the Human Rights Committee’s stipulation that states ‘enact a *protective* legal framework which includes effective *criminal* prohibitions on all manifestations of violence or incitement to violence that are likely to result in a deprivation of life’^108^ casts the penal apparatus as the foremost means of protecting human life across an array of circumstances.

The upending of what ought to be a default aversion (within democratic states and certainly within human rights institutions) to the employment of carceral tools can entail substantial harm to human dignity and human freedom, particularly if human rights penality is weaponised by security-driven states around the world. As Liora Lazarus has warned, the risk of coercive overreach emanating from the coercive orientation of positive obligations ‘may not lie … in the specific decisions of the courts, but rather in the reception of the messages in question within a broader politics of security’.^109^

While we may appreciate the immediate symbolic and expressive significance of ‘turning the cannons of the punitive machine against the powerful’,^110^ if penality is deemed the primary pathway for the protection of human rights, then the coercive suppression of human conduct may proliferate without (as much) challenge and with renewed righteousness. This is particularly threatening for the very persons that ‘progressive punitivism’^111^ sets out to protect: those on the margins of political goodwill, people who are already over-policed and under-protected by states’ carceral apparatus.^112^ The fault line in human rights penality’s egalitarian rationale appears particularly stark in this light.

Similarly troubling are human rights penality’s distorting implications for the human rights edifice itself. Human rights, seen in their best light, fundamentally promise—even if they might not, in their current form, bring about—emancipation and equality.^113^ Human rights demand human dignity-respecting treatment with which various typical manifestations of the criminal process (such as psychologically coercive interrogation ‘techniques’^114^) and of criminal punishment (such as prolonged solitary confinement^115^) are fundamentally incompatible. And human rights are increasingly interpreted as foreclosing or restricting certain punishments, such as the death penalty or life imprisonment without parole.^116^ There is, moreover, growing recognition within the human rights sphere that the retributivism of the penal sanction should be tempered—or, indeed, overridden—by the dignity-driven duty to provide meaningful pathways to rehabilitation for people in prison,^117^ not least in light of the fundamental human need for hope.^118^ There are thousands of authoritative findings of human rights abuses inflicted by states’ coercive and carceral apparatus, and regular acknowledgement of chronic and unyielding human rights violations within states’ carceral systems.^119^ In other words, human rights, in principle and in (much relevant) practice, tend in large part to stand for the sorts of things that penality antagonises.

As such, there is a striking disjunction between the counter-carcerality within human rights on the one hand and human rights penality on the other—a disjunction which at best complicates and at worst contradicts the effectiveness and equal protection rationales driving human rights penality, and which ultimately undermines the integrity of human rights. Concretely, the losses are already being counted. As Ailbhe O’Loughlin recently highlighted, the growing reliance and indeed pressure placed by human rights doctrine on the police and penal authorities to prevent serious violence has resulted in the effective construction of a ‘right to security’ capable of restricting rehabilitation opportunities^120^ and thereby undermining significant breakthroughs in the understanding of how imprisoned persons ought to be treated.^121^

### C. The Problem of Diversion

Recall the IACtHR’s statement:

The objective of international human rights law is not to punish those individuals who are guilty of violations, but rather to protect the victims and to provide for the reparation of damages resulting from the acts of the States responsible.^122^

This underlines that human rights penality should be understood as at most instrumental towards, and subsumed under, the overarching aims of effective—and equal—protection, reparation and accountability that (are meant to) shape human rights doctrine. Yet human rights penality has created a form of tunnel vision that diverts human rights actors from meaningful tools of protection, reparation and accountability beyond the penal frame.

In the domestic violence case of *Volodina v Russia*, for example, the ECtHR began its examination of Russia's fulfilment of the obligation to establish a protective legal framework by stating that ‘comprehensive legal and other measures are necessary to provide victims of domestic violence with effective protection and safeguards’.^123^ But it proceeded to focus primarily on the adequacy of criminal law provisions and criminal law enforcement in respect of domestic violence in Russia.^124^ This approach exemplifies the way in which the criminal law has become not only the *first* resort, but also often the *only* resort of the ECtHR in cases involving grave human rights violations. This tunnel vision, which has arisen out of an aim of bringing about ‘practical and effective protection’,^125^ serves in fact to sideline practical and effective measures of protection.^126^ In the context of domestic violence, the focus on policing, prosecution and punishment eclipses mechanisms enabling effective access to shelter, divorce, property, inheritance, financial security, child custody rights and related legal proceedings for (potential) victims/survivors,^127^ for example. Where non-penal measures are demanded, they often operate in the shadow and ‘orbit’, as Mattia Pinto puts it,^128^ of penality.^129^

The diversionary function of human rights penality is evident also in the construction of key legal instruments.^130^ In the United Nations Convention Against Torture, for example, it is possible to observe how the centring of the criminalisation of torture implicitly treats as peripheral other mechanisms by which torture can be effectively addressed and prevented. The Convention makes reference, but by no means accords primacy, to measures such as education, the review of interrogation practices and the facilitation of complaints, while other tools conducive to preventing torture, notably measures towards the alleviation of poverty,^131^ are conspicuously, if predictably, absent from its text.

Human rights penality, then, overshadows and thereby diverts us from other worthy tools of protection—tools that can be more effective than the mobilisation of penality in dismantling or alleviating the effects of, and systems and structures enabling, abuse and victimisation. The centrality of carcerality within human rights penality’s vision of effective and equal protection entails the disregard or dismissal of meaningful non-carceral or *counter-*carceral tools of protection. The importance of alleviating obstacles to protection, or other various law- and policy-grounded factors conducive to abuses, is also missed. Most relevant litigation involves little engagement, for example, with the importance of insulating mechanisms offering protection from violence from the apparatus of immigration control.^132^ The idea that the imperative of protection may even compel the *dismantling* of the carceral apparatus of immigration control as it stands in all too many parts of the world is hardly contemplated.^133^ The championing of criminal law ‘solutions’ against modern slavery and human trafficking, for example, has often gone hand in hand with aggressive anti-immigration policies and practices, as well as deregulatory approaches to labour rights.^134^ In this context, crime-control approaches prevail at the expense of the effective protection and empowerment of (potential) victims.

The label ‘diversion’ can capture both the opportunity costs *and* the limitations of human rights penality, and how these are obscured (or diverted from) by human rights penality’s tunnel vision. Consideration of the non-carceral avenues rendered peripheral by human rights penality can push us to interrogate the intuitive appeal of the effectiveness claim to which human rights penality tends to attach. The advance of human rights penality has—at least for some time—run parallel to a growth in critical insights destabilising long-held assumptions that the criminal justice system can effectively meet the broader needs of the community.^135^ There is a body of criminological and penological findings convincingly challenging the assumed efficacy of coercive and carceral tools in achieving the worthwhile ends with which they are often associated, notably the prevention of undesirable behaviour or the protection of vulnerable people.^136^ As former ECtHR Judge Françoise Tulkens has put it, ‘the effectiveness, particularly in terms of prevention, attributed to criminal law is a far cry from reality’.^137^ By way of example, relatively recent empirical studies on the subject have found that the criminal sanction appears to have no meaningful effect on recidivism for perpetrators of domestic violence.^138^ Human rights penality’s tunnel vision overlooks these insights, and not only assumes penality’s effectiveness in protecting people from grave harm, but casts penality as *the* most effective pathway to protection.^139^

Human rights penality’s capacity to divert also impacts on how redress and accountability are understood and concretised. In particular, human rights penality serves to decontextualise and restrict responses to alleged, suspected or confirmed human rights violations. This is palpable in how human rights bodies tend to delineate the duty to investigate human rights violations. Here, a focus on identifying and punishing those responsible^140^ unduly narrows the character and scope of the investigation required and accountability produced. As Karen Engle observes, penality shapes, and is shaped by, ‘an individualized and decontextualized understanding’ of the wrongdoing and harms involved.^141^ It can mean that human rights abuses are understood and treated as though they implicate lone individuals or groups acting aberrantly in isolated incidents, when it is widely observed that serious human rights violations are often part of broader systems or patterns of abuse, of which many are aware if not implicated, and which are often sustained by normative, institutional and structural enabling factors.^142^ In other words, an investigation that focuses on identifying and punishing the individuals responsible for the violation at issue (the so-called ‘bad apple(s)’^143^) can obscure, and in this way effectively absolve, the ‘rotten orchard’ (collective or institutional malpractice or discrimination, for example), as well as wider ecosystems of abuse: the structures that empower abusers and expose or themselves subject people to human rights violations.^144^ The investigatory focus on individual liability and punishment thereby misses key dimensions of human rights violations and denies the victims and wider ‘communities of harm’^145^ full reparation, including the establishment of truth and the implementation of effective guarantees of non-repetition.^146^

Accordingly, human rights penality compromises both the diagnosis and the cure when it comes to human rights violations, offering an impoverished account of accountability for serious wrongs, one which may obscure, ignore or even absolve institutions, systems and structures that inflict and foster abuse. As Silvana Tapia Tapia highlights, championing the criminal suppression of violence against women and consequently sidelining meaningful material protections can ultimately help uphold a neoliberal and fundamentally inegalitarian status quo.^147^ In this way, ‘the state’s abandonment of women is masked’^148^ and more transformative pathways to protection are precluded. Human rights penality’s tendency to divert attention from important dimensions of the human rights violation at issue can therefore serve at least implicitly to uphold or legitimise hollow or harmful state policies and practices, from aggressive immigration control to the dismantling of welfare guarantees. By rendering peripheral or even irrelevant the often pervasive factors that enable and perpetuate harm, including the conditions which shield the powerful while victimising and abandoning the powerless, human rights penality can therefore do the opposite of serving the effectiveness and equality rationales out of which it has emerged.

### D. The Hollowing Out of Visions of Justice

Linked to the observations above, human rights penality can have the effect of treating prosecution and the criminal sanction as not simply one mechanism towards, but rather as exhaustive of, ‘justice’ for human rights violations.^149^ This is at best a thin vision of justice, which elides the wider systemic and structural factors which enable and sustain both individual and multiple human rights abuses, and robs human rights of a more transformative vision of justice for human rights violations. At worst, it is a reactionary, damaging vision of justice, which fosters further abuses in justice’s name.

The idea that juridical justice, particularly through the criminal process, amounts to ‘thin’ justice has become well embedded in the field of transitional justice. A landmark intervention by Kieran McEvoy highlighted that the legalistic and narrow focus dominating the field displaced a more nuanced and open stance on transitional justice, foreclosing important questions and deprioritising key dimensions of the experience and normative treatment of societal transition from conflict and atrocity.^150^ Prominent among these matters is the tension between the pursuit of retributive justice on the one hand and endeavours that prioritise reconciliation and future stability on the other.^151^ Ruti Teitel observes that ‘individuation through litigation’ suppresses the more collective dimensions of justice in transitional contexts, notably diverting attention and resources from the pursuit of social justice.^152^ Indeed, Eric Stover finds that victims of atrocity often do not share the narrow vision of justice mediated through human rights penality’s retributive lens, but themselves highlight the importance of (a broad conception of) *social* justice. On their account, justice has to include

an array of social and economic rights … including the right to live … and to move about freely …; the right to have the bodies of loved ones returned for proper burial; the right to meaningful and secure jobs; and the right to receive adequate treatment for … psychological trauma.^153^

More fundamentally, Makau Mutua asks:

What kind of a lasting or effective solution would only focus on criminal sanctions for perpetrators while leaving completely unattended the moral and material needs of vulnerable individuals and groups in society? Would that not simply leave intact the power structures of yesterday and the fault lines that caused the pogroms in the first place?^154^

The imperative of transforming the power structures shaping the relevant abuses has been a growing preoccupation of those working in the field of transitional justice. Writing in 2009, Rodrigo Uprimny Yepes observed that, given the marginalisation and disadvantage faced by victims of serious human rights violations prior to, and indeed contributing to, the abuse to which they were subjected, reparations that sought to achieve restitution—that is, to restore them to the *status quo ante*—would be both unjust and contrary to the aim of seeking the non-repetition of such abuses.^155^ Accordingly, for Uprimny Yepes, reparations for mass human rights violations in unequal societies should ‘transform’ the unjust circumstances in which victims lived, which undoubtedly contributed to exposing them to further harm.^156^

The idea of ‘transformative reparations’ has received increasing attention in scholarship and activism, notably in relation to violence against women.^157^ As Rashida Manjoo has highlighted,^158^ ‘[since] violence perpetrated against individual women generally feeds into patterns of pre-existing and often cross-cutting structural subordination and systemic marginalisation, measures of redress need to link individual reparation and structural transformation’.^159^ She argued that ‘reparations should aspire, to the extent possible, to subvert instead of reinforce pre-existing patterns of crosscutting structural subordination, gender hierarchies, systemic marginalisation and structural inequalities that may be at the root cause of the violence that women experience’.^160^ While the idea of transformative reparations has chiefly taken hold in contexts of transition from conflict and mass atrocity, there is ample scope and good reason for treating transformative reparations as being relevant and applicable to any serious human rights violations that are embedded in, and shaped by, systemic and structural discrimination, marginalisation and injustice.^161^ Moreover, transformative reparations can be viewed as part of a broader concept: that of transformative justice, which may be understood to have at its heart the ‘challenging of unequal and intersecting power relationships and structures of exclusion at both local and global levels’.^162^ Transformative justice, which again need not be confined to post-conflict or otherwise rigidly bounded contexts, demands close attention to the systemic and structural dynamics that shape serious wrongdoing.

There may be a profound sense of justice in punishing persons who have wielded their relative power over others’ relative powerlessness in brutal ways. Yet human rights penality’s vision of justice can leave too much out, and too *many* out, particularly if the way they have been harmed is not ‘caught’ by the criminal law or criminal process. Even where prosecutions and punishments occur, human rights penality serves at best to redistribute punishment, rather than to redistribute resources in a way that meaningfully alters the systemic and structural factors that sustain the profound asymmetries of power and regard that anti-impunity campaigns seek to counter. To the extent that it *diverts* human rights actors from meaningfully transformative and redistributive interventions, human rights penality is not only limited, but also limiting: it hollows out prevailing visions of justice for past and future victims of human rights violations.

So much for the limitations and diversionary costs of human rights penality’s prioritisation of carceral justice. Yet framing justice for human rights violations in chiefly penal terms can also be challenged on a more wholesale basis. There are long-standing critiques of the inhumanity of punishment—in effect, retaliation—conceived as justice,^163^ particularly when meted out in a socially unjust world.^164^ Critics of ‘carceral feminism’—that is, of the alliance between feminist activists and the carceral state^165^—point to the ways in which this alliance, far from offering meaningful protection or justice, can fail or even threaten the safety and personal integrity of people experiencing (intersections of) disadvantage and marginalisation, and can serve to strengthen institutions that perpetuate not only violence against already vulnerable persons, but also profound material inequality, economic injustice and white supremacy.^166^ Recently, in a US-based initiative on police and prison abolition, Colin Kaepernick called the justice sought through criminal law tools, including in calls to prosecute and punish ‘killer cops’, ‘reactionary “justice”’, and argued that a ‘focus on individual punishment will never alter the outcome of a system rooted in Black death’.^167^ The idea of reconfiguring carceral tools towards more (nominally) just outcomes amounts, for abolitionists like Kaepernick, to tinkering with the machinery of Black death.^168^

On these accounts, in contexts where it is so indelibly tied to inequality and violence, carceral ‘justice’ must be outright *rejected* in favour of—rather than treated as complementary to—more reparative measures and more socially transformative pathways to effective and equal protection, empowerment, accountability and justice. But even if one is not prepared to view such wholesale rejection of carceral responses as a viable option for human rights institutions in the here and now, the testimony of those for whom carceral ‘justice’ means discriminatory violence and persecution must be heeded and responded to in reshaping the penal paradigm that currently prevails in the delineation of obligations to protect from, and redress, human rights violations.

## E. The Delegitimisation of Progressive Agendas

Human rights tend to be understood as a vision of ‘the good’, and compliance with human rights is widely treated as a criterion of legitimacy in public governance. In light of this, as alluded to above, the centring of penality in the fulfilment of human rights carries important legitimising qualities. In other words, human rights penality legitimises penality.^169^ Distilling protection, reparation and accountability into criminalisation, criminal law enforcement and criminal punishment can feed the narratives of nominally protective carceral initiatives even as these all too often harm even those whom they purport to protect.^170^

Importantly, the flip side of this is that human rights penality can serve to *de*legitimise counter-carceral idea(l)s. The idea of police and prison abolition has been richly theorised and vigorously pursued by feminist, queer, decolonial and other progressive counter-carceral thinkers and activists.^171^ In the aftermath of the killing of George Floyd, visions of police and prison abolition have been gaining more traction within progressive movements for equality around the world,^172^ and becoming the subject of an increasingly more concretised (re)imagination of pathways to protecting people from, and pursuing justice in response to, violence and other human rights abuses.^173^ Yet human rights penality forecloses abolition. Progressive calls for radical overhaul or abolition of the police and the prison are rendered incompatible with what are currently seen as non-negotiable imperatives of human rights. If the police and prison are authoritatively deemed primary protectors of human rights, a future that involves neither is impossible to imagine within the human rights frame. This delegitimisation of abolitionist thought and action leaves little scope for imagining and building alternative, non-carceral or counter-carceral visions of equality and justice within human rights, and thereby undermines human rights’ radical emancipatory potential.^174^

## 5. Conclusion: A Call for Unlocking Human Rights

Human rights penality is rationalised with reference to the equal and effective protection of rights, and the need to ensure meaningful accountability for their violation. Yet human rights penality is revealed to be limited, *limiting* and often actively harmful in the pursuit of these ends.

By tending towards equating protection with deterrence, and accountability with individual punishment, human rights penality hems in human rights in the name of bolstering them. It valorises penal systems and structures that are frequently—or, some would say, fundamentally—ill-suited to achieving practical and effective protection from human rights abuses, including abuses that they themselves often inflict and sustain. In allying and aligning with the criminal law and penal apparatus, human rights penality can harm both its purported beneficiaries and the human rights edifice itself. By super-imposing a criminal culpability standard onto human rights, human rights bodies unwittingly threaten to undermine the stringency of norms to which states are held under human rights law, and risk regress to an unduly narrow understanding of human rights. Moreover, human rights penality challenges the integrity of human rights, creating profound tensions between the interventions made on the basis of human rights to soften the edge of the carceral state, and the coercive push, through human rights penality, to sharpen it.

Many situations in which human rights are violated also encompass highly culpable behaviour by the individual(s) involved; many other instances, even where serious harm has been caused, do not involve such behaviour, or need not involve such behaviour to amount to (serious) human rights violations. *Most* human rights violations, however, take place against a backdrop of unequal, disempowering or oppressive systems and structures that enable and sustain abuse and injustice. Human rights penality, and the resulting tendency to align certain human rights violations with criminal liability, can therefore simultaneously overreach and under-include: it has the capacity to take penal coercion too far, and at the same time to prevent human rights from going far enough. Crucially, human rights penality can obscure key elements of the problems it seeks to address and can divert from, or even delegitimise, solutions that might better address them. Ultimately, the tunnel vision of human rights penality can be obstructive to broader visions of protection, accountability *and* justice.

Given the limitations, dangers and harms of human rights penality, how might it be rethought? The first step in rethinking the penal paradigm in human rights lies in revisiting the imperatives of effective and equal protection, accountability and justice. The overarching imperative of effective and equal protection, if taken seriously, requires us to contemplate a reorientation in human rights law and doctrine, which places protection rather than deterrence at the forefront and sees redistributive, non-carceral and even counter-carceral measures, such as the provision of meaningful material support for (potential) victims and the disentangling of protective mechanisms from immigration control, given primacy in the delineation of states’ obligations.

The idea of accountability itself can also be rethought, not least by recasting the focus onto those powerful actors that are the primary duty bearers under human rights law: state authorities.^175^ Accountability for human rights violations may be understood as necessitating a range of positive obligations through which the abuses inflicted, facilitated, condoned or otherwise enabled by state authorities through their acts and omissions are meaningfully identified, *contextualised* and addressed in full. A retreat from the penal paradigm can do the opposite of letting the state off the hook—rather, it can be used to demand larger-scale, more systematic responses oriented not only at individual redress, but also at transforming—or undoing—the structural and systemic conditions in which violations occur.^176^

Ultimately, those of us who believe in human rights ought to set our sights towards richer visions of justice, which seek to recognise the full scale of the harms inflicted on individuals and communities and to transform the conditions in which their infliction is made possible or condonable. In light of the ways in which material deprivation and inequality dehumanise persons and expose them to abuse, thinking and acting transformatively in terms of justice for human rights violations means resisting the persistent relegation of socioeconomic rights to second-class status, and placing essential socioeconomic protections at the forefront of lasting solutions to human rights abuse and injustice.^177^

## Footnotes

1 See eg OHCHR, ‘Statement on the Protests against Systemic Racism in the United States’ (5 June 2020) <www.ohchr.org/EN/NewsEvents/Pages/DisplayNews.aspx?NewsID=25927>.

2 Human rights bodies often engage with such authorities to prevent them from committing rights abuses. See eg the work of the United Nations Subcommittee on Prevention of Torture, discussed in R Murray and others (eds), *The Optional Protocol to the UN Convention Against Torture* (OUP 2011).

3 See the overviews offered in K Engle, ‘Anti-Impunity and the Turn to Criminal Law in Human Rights’ (2015) 100 Cornell L Rev 1069; M Pinto, ‘Historical Trends of Human Rights Gone Criminal’ (2020) 42 Hum Rts Q 729; N Mavronicola and L Lavrysen, ‘Coercive Human Rights: Introducing the Sharp Edge of the European Convention on Human Rights’ in L Lavrysen and N Mavronicola (eds), *Coercive Human Rights: Duties to Mobilise the Criminal Law Under the ECHR* (Hart Publishing 2020); S Malby, *Criminal Theory and International Human Rights Law* (Routledge 2020).

4 N Mavronicola, ‘Redistributing Punishment: The Limited Vision of Coercive Human Rights’ (*EJIL: Talk!*, 23 October 2020).

5 Lavrysen and Mavronicola (eds), *Coercive Human Rights* (n 3).

6 The phrase often used is ‘over-policed and under-protected’ – taken from David Muir, a representative of senior Black Church Leaders, quoted in Home Office, *The Stephen Lawrence Inquiry: Report of an Inquiry by Sir William Macpherson of Cluny* (Cm 4262-1, 1999).

7 See eg the contributions in K Engle, Z Miller and DM Davis (eds), *Anti-Impunity and the Human Rights Agenda* (CUP 2016); the reflections in R Kapur, *Gender, Alterity and Human Rights: Freedom in a Fishbowl* (Edward Elgar 2018); and the critical discussions in Lavrysen and Mavronicola (eds), *Coercive Human Rights* (n 3), particularly ch 1, 7–11 and postscript. These are illustrative examples; much of the remaining body of critical work I am referring to is cited at various points across this article.

8 F Tulkens, ‘The Paradoxical Relationship between Criminal Law and Human Rights’ (2011) 9 JICJ 577.

9 See ibid, and the genealogy of the ‘shield’ and ‘sword’ characterisation in fn 5 of her article. See also A Clapham, ‘Human Rights and International Criminal Law’ in WA Schabas (ed), *The Cambridge Companion to International Criminal Law* (CUP 2016) 11. For a more critical examination of how the ‘shield’ itself becomes the ‘sword’, see S Tapia Tapia, *Feminism, Violence Against Women, and Law Reform: Decolonial Lessons from Ecuador* (Routledge 2022) ch 4.

10 In line with Silvana Tapia Tapia, I understand ‘penality’ as referring to the entire penal ‘complex’, including criminal laws, processes and sanctions: S Tapia Tapia, ‘Feminism and Penal Expansion: The Role of Rights‑Based Criminal Law in Post‑Neoliberal Ecuador’ (2018) 26 Feminist Legal Studies 285, 287. More broadly, ‘penality’ can be understood as ‘penality’ encompassing penal laws, sanctions, institutions, practices and *discourses*—see Pinto, ‘Historical Trends’ (n 3) fn 3; see further D Garland, *Punishment and Welfare: A History of Penal Strategies* (Gower 1985) x.

11 See the overviews offered in Pinto, ‘Historical Trends’ (n 3).

12 Convention Against Torture and Other Cruel, Inhuman or Degrading Treatment or Punishment, 1984, 1465 UNTS 85.

13 International Convention for the Protection of All Persons from Enforced Disappearances, 2006, 2716 UNTS 3. For an account of human rights treaties incorporating duties to mobilise the criminal law in respect of human rights violations, see A Seibert-Fohr, *Prosecuting Serious Human Rights Violations* (OUP 2009) ch 5.

14 See the critical analysis in N Mavronicola, ‘Coercive Overreach, Dilution and Diversion: Potential Dangers of Aligning Human Rights Protection with Criminal Law (Enforcement)’ in Lavrysen and Mavronicola (eds), *Coercive Human Rights* (n 3) 184.

15 See Pinto, ‘Historical Trends’ (n 3); see also Lavrysen and Mavronicola (eds), *Coercive Human Rights* (n 3); Engle, ‘Anti-Impunity’ (n 3).

16 See Seibert-Fohr (n 13) ch 3; FF Basch, ‘The Doctrine of the Inter-American Court of Human Rights Regarding States’ Duty to Punish Human Rights Violations and Its Dangers’ (2007) 23 Am U Int’l L Rev 195; F Mégret and J-P Saucier Calderón, ‘The Move Towards a Victim‑Centred Concept of Criminal Law and the “Criminalization” of Inter‑American Human Rights Law: A Case of Human Rights Law Devouring Itself?’ in Y Haeck, O Ruiz-Chiriboga and C Burbano Herrera (eds), *The Inter-American Court of Human Rights: Theory and Practice, Present and Future* (Intersentia 2015) 419.

17 See Pinto, ‘Historical Trends’ (n 3).

18 Lavrysen and Mavronicola (eds), *Coercive Human Rights* (n 3).

19 See eg *Volodina v Russia* App no 41261/17 (ECtHR, 9 July 2019) paras 78–85. The judgment in *Volodina* builds on a body of case law on domestic violence and Article 3 ECHR which prizes criminal law measures to prevent and respond to domestic violence. See notably *Opuz v Turkey* App no 33401/02 (ECtHR, 9 June 2009) paras 128–49, 158–76, 199–202; *Valiuliene v Lithuania* App no 33234/07 (ECtHR, 26 March 2013) paras 75–85. See also the emphasis on criminal law provisions in *O’Keeffe v Ireland* App no 35810/09 (ECtHR, 28 January 2014) para 148; *Siliadin v France* App no 73316/01 (ECtHR, 26 July 2005) para 112; *Rantsev v Cyprus and Russia* App no 25965/04 (ECtHR, 7 January 2010) para 218.

20
*Osman v UK* App no 87/1997/871/1083 (ECtHR, 28 October 1998) para 116.

21
*Đorđević v Croatia* App no 41526/10 (ECtHR, 24 July 2012) para 139. See also *Galotskin v Greece* App no 2945/07 (ECtHR, 14 January 2010) para 36. See similarly in relation to Article 2 ECHR, *Opuz* (n 19) para 129; *Edwards v UK* App no 46477/99 (ECtHR, 14 March 2002) para 55.

22
*O’Keeffe* (n 19) para 172.

23 See the discussion in Seibert-Fohr (n 13) chs 2–4.

24
*Fornerón and Daughter v Argentina* (IACtHR, 27 April 2012) para 140.

25
*Velásquez-Rodríguez v Honduras* (IACtHR, 29 July 1988) para 175. See the further analysis in L Lavrysen, ‘Positive Obligations in the Jurisprudence of the Inter-American Court of Human Rights’ (2014) 7 Int Am & Eur Hum Rts J 94, especially 101–3. On the ‘duty to punish’ in the IACtHR’s doctrine, see Basch (n 16).

26 See eg United Nations Human Rights Committee, ‘General Comment 36’, UN Doc CCPR/C/GC/36, para 20; United Nations Human Rights Committee, ‘General Comment 20’, UN Doc HRI/GEN/1/Rev.1, para 13.

27 On anti-impunity and human rights, see Engle, Miller and Davis (eds), *Anti-Impunity* (n 7). For an earlier discussion focused on combating impunity, see N Roht-Arriaza, ‘Combating Impunity: Some Thoughts on the Way Forward’ (1996) 59 Law & Contemporary Problems 93.

28 Seibert-Fohr (n 13) 1. For a critical discussion of this idea, see S Moyn, ‘Anti-Impunity as Deflection of Argument’ in Engle, Miller and Davis (eds), *Anti-Impunity* (n 7) 68.

29 See WA Schabas, ‘International Justice for International Crimes: An Idea Whose Time Has Come’ (2006) 14 European Review 421.

30 See WA Schabas, *Unimaginable Atrocities: Justice, Politics, and Rights at the War Crimes Tribunals* (OUP 2012) ch 1.

31 LA Payne, ‘The Justice Paradox? Transnational Legal Orders and Accountability for Past Human Rights Violations’ in TC Halliday and G Shaffer (eds), *Transnational Legal Orders* (CUP 2015) 443. On justice understood as being effected and ‘cascading’ through prosecutions, see K Sikkink, *The Justice Cascade: How Human Rights Prosecutions Are Changing World Politics* (WW Norton 2011). For critical reflections on the idea of ‘doing justice’ in the realm of international criminal justice, see S Nouwen and W Werner, ‘Doing Justice to the Political: The International Criminal Court in Uganda and Sudan’ (2011) 21 EJIL 941; S Nouwen, ‘Justifying Justice’ in J Crawford and M Koskenniemi (eds), *The Cambridge Companion to International Law* (CUP 2015) 327; B Sander, *Doing Justice to History: Confronting the Past in International Criminal Courts* (OUP 2021).

32 Seibert-Fohr (n 13) 55, citing Inter-American Commission of Human Rights, *Masacre Las Hojas v El Salvador*, report, 24 September 1992, 83; Inter-American Commission of Human Rights, *Carmelo Soria Espinoza v Chile*, report, 19 November 1999, para 66.

33
*Bulacio v Argentina* (IACtHR, 18 September 2003), para 115.

34
*Garrido and Baigorria v Argentina* (IACtHR, 27 August 1998), para 69.

35 ibid para 71 (emphasis added).

36
*Cestaro v Italy* App no 6884/11 (ECtHR, 7 April 2015) para 207.

37 L Lazarus, ‘Positive Obligations and Criminal Justice: Duties to Protect or Coerce?’ in L Zedner and J Roberts (eds), *Principles and Values in Criminal Law and Criminal Justice* (OUP 2012) 135.

38 P Lemmens and M Courtoy, ‘Positive Obligations and Coercion: Deterrence as a Key Factor in the European Court of Human Rights’ Case Law’ in Lavrysen and Mavronicola (eds), *Coercive Human Rights* (n 3) 57 (citations omitted).

39 ibid 55.

40
*X and Y v The Netherlands* App no 8978/80 (ECtHR, 26 March 1985) para 27 (emphasis added).

41
*Volodina* (n 19) para 86 (emphasis added); see also *Opuz* (n 19) para 176; *Rantsev* (n 19) para 218.

42 See text to n 26 above.

43 K Engle, Z Miller and DM Davis, ‘Introduction’ in Engle, Miller and Davis (eds), *Anti-Impunity* (n 7) 1.

44 M Bovens, ‘Analysing and Assessing Accountability: A Conceptual Framework’ (2007) 13(4) ELJ 447, 448.

45 For an insightful critical analysis of the concept of ‘serious’ human rights violations and its implications, see I Siatitsa, *Serious Violations of Human Rights: On the Emergence of a New Special Regime* (OUP 2022).

46 Payne (n 31) 439. See also Seibert-Fohr (n 13) 189.

47 Consider eg T Meron, ‘Closing the Accountability Gap: Concrete Steps towards Ending Impunity for Atrocity Crimes’ (2018) 112(3) AJIL 433; J E. Méndez and J Mariezcurrena, ‘Accountability for Past Human Rights Violations: Contributions of the Inter-American Organs of Protection’ (1999) 26(4) Social Justice 84. The nuances involved are acknowledged in A Devereux, ‘Accountability for Human Rights Violations in Syria: Reasons for Hope’ (2019) 25(3) AJHR 391, 393.

48 See eg *O’Keeffe* (n 19) para 172.

49
*Öneryildiz v Turkey* App no 48939/99 (ECtHR, 30 November 2004) para 116.

50 ibid para 117.

51 This is reflected in the analysis in Méndez and Mariezcurrena (n 47).

52
*Paniagua-Morales and others v Guatemala* (IACtHR, 8 March 1998) para 173.

53 Human Rights Committee, ‘General Comment 36’ (n 26) para 27.

54 ibid.

55 One of the most notable accounts aligning criminal accountability with ‘justice’ is Kathryn Sikkink’s account of the ‘justice cascade’: see Sikkink (n 31).

56 ibid 8.

57 K McEvoy and L Mallinder, ‘Amnesties in Transition: Punishment, Restoration, and the Governance of Mercy’ (2012) 39 Journal of Law and Society 410; LA Payne, F Lessa and G Pereira, ‘Overcoming Barriers to Justice in the Age of Human Rights Accountability’ (2015) 37(3) Hum Rts Q 728. See the nuanced analysis in V Spiga, ‘No Redress without Justice: Victims and International Criminal Law’ (2012) 10 JICJ 1377.

58 See the critique of such a narrow treatment of even *criminal* justice in M Pensky, ‘Amnesty on Trial: Impunity, Accountability, and the Norms of International Law’ (2008) 1(1–2) Ethics & Global Policy 1.

59 C Heri, ‘Shaping Coercive Obligations through Vulnerability: The Example of the ECtHR’ in Lavrysen and Mavronicola (eds), *Coercive Human Rights* (n 3) 93.

60 ibid.

61 S Stavros, ‘Criminal Law Responses to Hate Speech: Towards a Systematic Approach in Strasbourg?’ in Lavrysen and Mavronicola (eds), *Coercive Human Rights* (n 3) 119.

62 ibid 138.

63 Heri (n 59) 115.

64 A Balta, ‘Retribution through Reparations? Evaluating the European Court of Human Rights’ Jurisprudence on Gross Human Rights Violations from a Victim’s Perspective’ in Lavrysen and Mavronicola (eds), *Coercive Human Rights* (n 3) 71.

65 See text to n 53 above.

66 The phrase is taken from a participant in the Stephen Lawrence murder inquiry – see n 6 above. See further L Wacquant, *Punishing the Poor: The Neoliberal Government of Social Insecurity* (Duke UP 2009).

67 H Aviram, ‘Progressive Punitivism: Notes on the Use of Punitive Social Control to Advance Social Justice Ends’ (2020) 68 Buff L Rev 199, 201.

68 ibid.

69 See eg P Lenta, ‘Transitional Justice and Retributive Justice’ (2019) 22 Ethical Theory and Moral Practice 385, 392–3; Balta (n 64) 78ff; but see a critique of retributive justice in transitional contexts in C Murphy, *The Conceptual Foundations of Transitional Justice* (CUP 2017) 91–6.

70 The issue of alignment or conflation is discussed and critiqued in, among others, D Robinson, ‘The Identity Crisis of International Criminal Law’ (2008) 21 LJIL 925, 929, 946–9; V Stoyanova, ‘Article 4 of the ECHR and the Obligation of Criminalising Slavery, Servitude, Forced Labour and Human Trafficking’ (2014) 3 CJICL 407, 441.

71 See the related argument in BR Roth, ‘Just Short of Torture: Abusive Treatment and the Limits of International Criminal Justice’ (2008) 6 JICJ 215, 235.

72 United Nations Special Rapporteur on Torture, ‘Thematic Report: Commemorating 70th Anniversary of the Universal Declaration of Human Rights’ (20 July 2018) UN Doc A/73/207, para 7.

73 ibid; United Nations Special Rapporteur on Torture, ‘Thematic Report: Study on the Phenomena of Torture, Cruel, Inhuman or Degrading Treatment or Punishment in the World’ (5 February 2010) UN Doc A/HRC/13/39/Add.5, para 188; see also *MSS v Belgium and Greece* App no 30696/09 (ECtHR, 21 January 2011) para 220.

74 See notably *Vinter v UK* App no 66069/09 (ECtHR, 9 July 2013). See the discussion of the significance of *Vinter* in D Van Zyl Smit and C Appleton, *Life Imprisonment: A Global Human Rights Analysis* (Harvard UP 2019) ch 11.

75 See Human Rights Committee, *Mellet v Ireland* (17 November 2016) UN Doc CCPR/C/116/D/2324/2013.

76 See the cumulatively and/or systemically degrading conditions of detention in cases such as *Peers v Greece* (2001) 33 EHRR 1192; *Kalashnikov v Russia* (2003) 36 EHRR 34; *Orchowski v Poland* App no 17885/04 (ECtHR, 22 October 2009).

77 See further N Mavronicola, ‘Taking Life and Liberty Seriously: Reconsidering Criminal Liability under Article 2 of the ECHR’ (2017) 80(6) MLR 1026.

78 See ibid 1029–30.

79
*McCann v UK* App no 18984/91 (ECtHR, 27 September 1995) para 149.

80 See eg *Giuliani and Gaggio v Italy* App no 23458/02 (ECtHR, 24 March 2011) para 178.

81
*Da Silva v UK* App no 5878/08 (ECtHR, 30 March 2016) paras 245–8.

82 ibid paras 244–56.

83 ibid para 248.

84 See S Dothan, ‘The Three Traditional Approaches to Treaty Interpretation: Current Application to the European Court of Human Rights’ (2019) 42(3) Fordham Int’l LJ 765, 768; S Dothan, ‘In Defence of Expansive Interpretation in the European Court of Human Rights’ (2014) 3(2) CJICL 508. For an illuminating take on expansive interpretations of human rights, see J Theilen, ‘The Inflation of Human Rights: A Deconstruction’ (2021) 34(4) LJIL 831.

85
*Nicolae Virgiliu Tănase v Romania* App no 41720/13 (ECtHR, 25 June 2019) para 121.

86 ibid Partly Dissenting Opinion of Judge Kūris, para 5.

87
*YP v Russia* App no 43399/13 (ECtHR, 20 September 2022) paras 31–8. Note the Partly Dissenting Opinions by Judge Serghides and Judge Pavli, and the critical reflections in Z Tongue and L Graham, ‘YP v Russia: Sterilisation Without Consent, Article 3, and Weak Reproductive Rights at the ECtHR’ (*Strasbourg Observers*, 30 September 2022) <https://strasbourgobservers.com/2022/09/30/y-p-v-russia-sterilisation-without-consent-article-3-and-weak-reproductive-rights-at-the-ecthr/>.

88
*YP* (n 87) para 37.

89 ibid Concurring Opinion of Judge Elósegui, para 13.

90 ibid para 14.

91 ibid para 17.

92 This would be contrary to Article 3 ECHR according to the ECtHR in *Vinter* (n 74); *Murray v Netherlands* App no 10511/10 (ECtHR, 25 April 2016).

93 See, eg, *Orchowski* (n 76)—and the discussion in Linos-Alexandre Sicilianos, ‘The European Court of Human Rights at a time of crisis in Europe’ (2016) 2 EHRLR 121.

94 See eg *MSS* (n 73).

95 A notable illustration of such an argument can be found in John Finnis’s attempt to challenge the *non-refoulement* duty under Article 3 ECHR and equivalent provisions in other instruments: see J Finnis, ‘Absolute Rights: Some Problems Illustrated’ (2016) 61(2) Am J Juris 195.

96 See eg *Ireland v UK* App no 5310.71 (ECtHR, 18 January 1978) para 161; *Finogenov v Russia* App nos 18299/03 and 27311/03 (ECtHR, 20 December 2011) para 199.

97 On the standard of ‘beyond reasonable doubt’ in criminal law, see A Keane and P McKeown, *The Modern Law of Evidence* (13th edn, OUP 2020) ch 3.

98 In this regard, see *Tomasi v France* App no 12850/87 (ECtHR, 27 August 1992) paras 108–11; *Ribitsch v Austria* App no 18896/91 (ECtHR, 4 December 1995) para 34; *Diri v Turkey* App no 68351/01 (ECtHR, 31 July 2007) paras 35–9; but cf *Klaas v Germany* App no 15473/89 (ECtHR, 22 September 1993).

99 For a critical overview, see C Roberts, ‘Reversing the Burden of Proof before Human Rights Bodies’ (2021) 25(10) International Journal of Human Rights 1682. On the Inter-American system, see A Bovino, ‘Evidential Issues Before the Inter-American Court of Human Rights’ (2005) 2(3) Sur Journal 57. On fact-finding missions and inquiries, see S Wilkinson, *Standards of Proof in International Humanitarian and Human Rights Fact-Finding and Inquiry Missions* (Geneva Academy Report 2012).

100
*Velásquez-Rodríguez* (n 25) paras 134-5; *Godínez-Cruz v Honduras* (IACtHR, 20 January 1989) paras 140–1.

101 See eg *Khatuyeva and others v Russia* App nos 4310/10, 75318/11 and 71377/13 (ECtHR, 27 August 2019) para 115; *Salikhova and Magomedova v Russia* App no 63689/13 (ECtHR, 26 January 2016) paras 65–72; *Cadıroğlu v Turkey* App no 15762/10 (ECtHR, 3 September 2013) paras 24–7; *Adali v Turkey* App no 38187/97 (ECtHR, 31 March 2005) paras 211–20.

102 M Möschel, ‘Is the European Court of Human Rights’ Case Law on Anti-Roma Violence “Beyond Reasonable Doubt”?’ (2012) 12(3) HRLR 479; R Rubio-Marín and M Möschel, ‘Anti-Discrimination Exceptionalism: Racist Violence before the ECtHR and the Holocaust Prism’ (2015) 26(4) EJIL 881. See also M-B Dembour, ‘In the Name of the Rule of Law: The European Court of Human Rights’ Silencing of Racism’ in G K Bhambra and R Shilliam (eds), *Silencing Human Rights: Critical Engagements with a Contested Project* (Palgrave Macmillan 2009) 184.

103 N Peršak, ‘Positive Obligations in View of the Principle of Criminal Law as a Last Resort’ in Lavrysen and Mavronicola (eds), *Coercive Human Rights* (n 3) 141. See also Basch (n 16) 215.

104 N Jareborg, ‘Criminalization as Last Resort (*Ultima Ratio*)’ (2005) 2 Ohio State Journal of Criminal Law 521; N Jareborg, ‘What Kind of Criminal Law Do We Want?’ in N Jareborg (ed), *Scraps of Penal Theory* (Iustus Forlag 2002). Note the more critical take in D Husak, ‘The Criminal Law as Last Resort’ (2004) 24(2) OJLS 207.

105 See the ECtHR’s statement in *Pretty v UK* App no 2346/02 (ECtHR, 29 April 2002), para 65. The Preamble of the Universal Declaration of Human Rights also centres human dignity and human freedom—see UN General Assembly, Universal Declaration of Human Rights, 217A(III), 10 December 1948.

106 See eg *Velásquez-Rodriguez* (n 25) para 176.

107 See eg *Volodina* (n 19) paras 76–101; *Talpis v Italy* App no 41237/14 (ECtHR, 2 March 2017) paras 100–31; *Opuz* (n 19) paras 128–76. See the critical discussion in K Engle, ‘Abolitionism and the European Court of Human Rights: Resonances and Dissonances in Approaches to Human Rights’ (on file with author).

108 United Nations Human Rights Committee, ‘General Comment 36’ (n 26) para 20 (emphasis added).

109 Lazarus (n 37) 141.

110 Aviram (n 67) 201.

111 ibid.

112 See eg the discussion in S Lamble, ‘Queer Investments in Punitiveness: Sexual Citizenship, Social Movements and the Expanding Carceral State’ in J Haritaworn, A Kuntsman and S Posocco (eds), *Queer Necropolitics* (Routledge 2014).

113 But see the sustained critical examination of such ideas in Kapur (n 7).

114 See eg *Gäfgen v Germany* App no 22978/05 (ECtHR, 1 June 2010).

115 See eg United Nations Special Rapporteur on Torture, ‘Thematic Report: Solitary Confinement’ (5 August 2011) UN Doc A/66/268.

116 In this respect, see WA Schabas, *The Abolition of the Death Penalty in International Law* (3rd edn, CUP 2002); Van Zyl Smit and Appleton (n 74).

117 See *Vinter* (n 74); see also the analysis provided in S Meijer, ‘Rehabilitation as a Positive Obligation’ (2017) 25 Eur J Crime Cr L Cr J 145.

118 See the analysis in *Vinter* (n 74), Concurring Opinion of Judge Power-Forde; see also K Brownlee, ‘Punishment and Precious Emotions: A Hope Standard for Punishment’ (2021) 41(3) OJLS 589.

119
*Orchowski* (n 76) para 153—noted by Judge Linos-Alexandre Sicilianos as being indicative of an obligation ‘of result’ rather than of means: Sicilianos (n 93) 124.

120 A O’Loughlin, ‘Risk Reduction and Redemption: An Interpretive Account of the Right to Rehabilitation in the Jurisprudence of the European Court of Human Rights’ (2021) 41(2) OJLS 510.

121 See the discussion of Article 3 standards on punishment and imprisonment in N Mavronicola, ‘Crime, Punishment and Article 3 ECHR: Puzzles and Prospects of Applying an Absolute Right in a Penal Context’ (2015) 15 HRLR 721, which includes an assessment of the significance of *Vinter* (n 74).

122
*Velásquez-Rodriguez* (n 25) para 134.

123
*Volodina* (n 19) para 78.

124 ibid paras 78–85.

125
*Siliadin* (n 19) para 148.

126 A limited critique in this respect, focusing specifically on the limitations of the dominant approach to the operational positive duty under the right to life, can be found in FC Ebert and R I Sijniensky, ‘Preventing Violations of the Right to Life in the European and the Inter-American Human Rights Systems: From the *Osman* Test to a Coherent Doctrine on Risk Prevention?’ (2015) 15 HRLR 343.

127 In this regard, see L Williams and S Walklate, ‘Policy Responses to Domestic Violence, the Criminalisation Thesis and “Learning from History”’ (2020) 59(3) The Howard Journal of Crime and Justice 305.

128 Pinto, ‘Sowing a “Culture of Conviction”: What Shall Domestic Criminal Justice Systems Reap from Coercive Human Rights?’ in Lavrysen and Mavronicola (eds), *Coercive Human Rights* (n 3) 170.

129 It is instructive here to mention a less obvious example: in *Rantsev* (n 19), the ECtHR called for the revocation of Cyprus’s ‘artiste’ visa regime, which the Court identified as facilitating trafficking: in doing so, it focused on how this regime impeded criminal law enforcement, rather than on how it interacted with a carceral system of immigration control that vulnerabilised (prospective) victims.

130 See the analysis of some relevant instruments in Pinto, ‘Historical Trends’ (n 3) 738–41.

131 On the links between torture and poverty, see TE McCarthy (ed), *Attacking the Root Causes of Torture: Poverty, Inequality and Violence* (OMCT 2006); S Jensen and M Koch Andersen (eds), *Corruption and Torture: Violent Exchange and the Policing of the Urban Poor* (Aalborg UP 2017).

132 For a critical examination of such protection in the context of the right not to be subjected to slavery or servitude, see V Stoyanova, ‘Separating Protection from the Exigencies of the Criminal Law: Achievements and Challenges under Article 4 ECHR’ in Lavrysen and Mavronicola (eds), *Coercive Human Rights* (n 3) 203.

133 Nonetheless, both the United Nations Special Rapporteur on Torture and the United Nations Special Rapporteur on Extrajudicial Killings have challenged a range of restrictive anti-immigration policies and practices in recent reports—for example, the Torture Rapporteur has said: ‘The primary cause for the massive abuse suffered by migrants in all regions of the world, including torture, rape, enslavement, trafficking and murder, is neither migration itself, nor organized crime, nor the corruption of individual officials, but the growing tendency of States to base their official migration policies and practices on deterrence, criminalization and discrimination rather than on protection, human rights and non-discrimination’— United Nations Special Rapporteur on Torture, ‘Thematic Report: Migration-related Torture and Ill-treatment’ (23 November 2018) UN Doc A/HRC/37/50, para 66. See also United Nations Special Rapporteur on Extrajudicial, Summary or Arbitrary Executions, ‘Thematic Report: Unlawful Death of Refugees and Migrants’ (15 August 2017) UN Doc A/72/335.

134 See E Kenway, *The Truth About Modern Slavery* (Pluto Press 2020); J Christman, ‘Human Rights and Global Wrongs: The Role of Human Rights Discourse in Responses to Trafficking’ in D Tietjens Meyers (ed), *Poverty, Agency, and Human Rights* (OUP 2014); M Pinto, ‘Discursive Alignment of Trafficking, Rights and Crime Control’ (2023) 19 International Journal of Law in Context 122.

135 See eg SL Hulsman, ‘Critical Criminology and the Concept of Crime’ (1986) 10 Contemporary Crises 63, 64; Y Russell, ‘Criminal Injustice’ in I Rua Wall and others (eds), *The Critical Legal Pocketbook* (Counterpress 2021).

136 European Committee on Crime Problems, *Report on Decriminalisation* (Council of Europe 1980) 75–8. See the discussion of this report in M Pinto, ‘Coercive Human Rights and the Forgotten History of the Council of Europe’s *Report on Decriminalisation*’ (2023) 86(5) MLR 1108. See also L Sherman, ‘Defiance, Deterrence, and Irrelevance: A Theory of the Criminal Sanction’ (1993) 30(4) Journal of Research in Crime and Delinquency 445.

137 F Tulkens, ‘Human Rights as the Good and the Bad Conscience of Criminal Law’ in S Snacken and E Dumortier (eds), *Resisting Punitiveness in Europe? Welfare, Human Rights and Democracy* (Routledge 2011) 158.

138 FA Sloan, AC Platt, LM Chepke and CE Blevins, ‘Deterring Domestic Violence: Do Criminal Sanctions Reduce Repeat Offenses?’ (2013) 46 Journal of Risk and Uncertainty 51. Note also L Goodmark, *Decriminalizing Domestic Violence: A Balanced Policy Approach to Intimate Partner Violence* (University of California Press 2018) 146.

139 See the wide-ranging critical treatment of this in A Sattar, *Criminal Punishment and Human Rights: Convenient Morality* (Routledge 2019).

140 See text to n 22.

141 Engle, ‘Anti-Impunity’ (n 3) 1071.

142 See eg D Celermajer, *The Prevention of Torture: An Ecological Approach* (CUP 2018).

143 M Punch, ‘Rotten Orchards: “Pestilence”, Police Misconduct and System Failure’ (2003) 13 Policing and Society 171.

144 See further S Marks, ‘Human Rights and Root Causes’ (2011) 74(1) MLR 57.

145 See F Ni Aolain, ‘Sex-based Violence during the Holocaust—A Reevaluation of Harms and Rights in International Law’ (2000) 12 Yale J L Feminism 43. For rich analysis of the (bounded) recognition of communities of harm and reparations, see: R Rubio-Marín, C Sandoval and C Díaz, ‘Repairing Family Members: Gross Human Rights Violations and Communities of Harm’ in R Rubio-Marín (ed), *The Gender of Reparations: Unsettling Sexual Hierarchies while Redressing Human Rights Violations* (CUP 2009); D Gonzalez-Salzberg, ‘Queering Reparations Under International Law: Damages, Suffering, and (Heteronormative) Kinship’ (2022) 116 AJIL Unbound 5.

146 Guarantees of non-repetition are identified as a key form of reparation or gross violations of human rights in the United Nations Basic Principles and Guidelines on the Right to a Remedy and Reparation for Victims of Gross Violations of International Human Rights Law and Serious Violations of International Humanitarian Law, adopted and proclaimed by General Assembly resolution 60/147 of 16 December 2005 (UN Doc A/RES/60/147), Articles 18 and 23.

147 On the alliance between neoliberalism and carcerality, see Tapia Tapia, ‘Feminism and Penal Expansion’ (n 10) 287. But see her interrogation of ‘post-neoliberal’ penality and human rights thereafter.

148 S Tapia Tapia, ‘Beyond Carceral Expansion: Survivors’ Experiences of Using Specialised Courts for Violence Against Women in Ecuador’ (2021) 30(6) Social and Legal Studies 848.

149 See Moyn (n 28).

150 K McEvoy, ‘Beyond Legalism: Towards a Thicker Understanding of Transitional Justice’ (2007) 34 Journal of Law and Society 411. See further N Palmer, P Clark and D Granville (eds), *Critical Perspectives in Transitional Justice* (Intersentia 2012).

151 M Jackson, ‘Amnesties in Strasbourg’ (2018) 38(3) OJLS 451, 467.

152 R Teitel, ‘Transitional Justice and Judicial Activism—A Right to Accountability’ (2015) 48(2) Cornell Int’l LJ 385, 420. See also EA Fattah, ‘Is Punishment the Appropriate Response to Gross Human Rights Violations? Is a Non-punitive Justice System Feasible?’ (2007) Acta Juridica 209.

153 E Stover, *The Witnesses: War Crimes and the Promise of Justice in The Hague* (University of Pennsylvania Press 2005) 119–20.

154 M Mutua, ‘A Critique of Rights in Transitional Justice: The African Experience’ in G Oré Aguilar and F Gómez Isa (eds), *Rethinking Transitions: Equality and Social Justice in Societies Emerging from Conflict* (Intersentia 2011) 44.

155 R Uprimny Yepes, ‘Transformative Reparations of Massive Gross Human Rights Violations: Between Corrective and Distributive Justice’ (2009) 27 NQHR 625. On guarantees of non-repetition as a component of reparations for gross human right violations, see UN ‘Basic Principles’ (n 146) Articles 18 and 23.

156 Uprimny Yepes (n 155) 637–8.

157 See eg Rubio-Marín (ed), *The Gender of Reparations* (n 145) notably ch 3 (by C Duggan and R Jacobson) and ch 8 (by R Rubio-Marín); C Duggan and AM Abusharaf, ‘Reparation of Sexual Violence in Democratic Transitions: The Search for Gender Justice’ in P de Greiff (ed), *The Handbook of Reparations* (OUP 2006). See also Coalition for Women’s Human Rights in Conflict Situations and others, ‘Nairobi Declaration on Women’s and Girls’ Right to a Remedy and Reparation’ (International Meeting on Women’s and Girls’ Right to a Remedy and Reparation, Nairobi, 19–21 March 2007), para 3.

158 United Nations Special Rapporteur on Violence against Women, Its Cases and Consequences, ‘Thematic Report: Reparations for Women Subjected to Violence’ (23 April 2010) UN Doc A/HRC/14/22, para 23.

159 ibid para 24.

160 ibid para 85. See further R Manjoo, ‘Introduction: Reflections on the Concept and Implementation of Transformative Reparations’ (2017) 21(9) International Journal of Human Rights 1193. For an examination of some of the transformative scope of the IACtHR's reparations jurisprudence, see R Rubio-Marín and C Sandoval, 'Engendering the Reparations Jurisprudence of the Inter-American Court of Human Rights: The Promise of the *Cotton Field* Judgment' (2011) 33(4) Human Rights Quarterly 1062.

161 See N Mavronicola and L Davies, ‘“Conversion Therapy” and Transformative Reparations’ in I Trispiotis and C Purshouse (eds), *Banning ‘Conversion Therapy’: Legal and Policy Perspectives* (Hart Publishing 2023) 241.

162 P Gready and S Robins, ‘From Transitional to Transformative Justice: A New Agenda for Practice’ in P Gready and S Robins (eds), *From Transitional to Transformative Justice* (Cambridge, Cambridge UP 2019) 32.

163 See eg HE Barnes, *The Story of Punishment: A Record of Man’s Inhumanity to Man* (Patterson Smith 1931).

164 See eg the argument regarding the unacceptability of imposing punishment in contexts of serious social injustice and exclusion in RA Duff, *Punishment, Communication, and Community* (OUP 2001), discussed in J Holroyd, ‘Punishment and Justice’ (2010) 36 Social Theory and Practice 78.

165 See E Bernstein, ‘The Sexual Politics of the New Abolitionism’ (2007) 18(3) Differences 128. See also E Bernstein, ‘Carceral Politics as Gender Justice? The “Traffic in Women” and Neoliberal Circuits of Crime, Sex, and Rights’ (2012) 41 Theory and Society 233. See also A Gruber, ‘The Feminist War on Crime’ (2007) 92 Iowa L Rev 741; A Gruber, ‘A “Neo-Feminist” Assessment of Rape and Domestic Violence Law Reform’ (2012) 15 Journal of Gender, Race and Justice 583. Note the criticism of ‘anti-carceral feminism’ in C McGlynn, ‘Challenging Anti-carceral Feminism: Criminalisation, Justice and Continuum Thinking’ (2022) 93 Women’s Studies International Forum 102614.

166 B E Richie, *Arrested Justice: Black Women, Violence, and America’s Prison Nation* (NYU Press 2012); E Boodman, ‘An Immanent Critique of the Prison Nation: The Contradictions of Carceral “Anti-violence”’ (2018) 44(5) Philosophy and Social Criticism 571; M Pérez and B Radi, ‘Gender Punitivism: Queer Perspectives on Identity Politics in Criminal Justice’ (2020) 20(5) Criminology & Criminal Justice 523. See also the landmark piece by Kay Harris: MK Harris, ‘Moving into the New Millennium: Toward a Feminist Vision of Justice’ (1987) 67 Prison Journal 27.

167 C Kaepernick, ‘The Demand for Abolition’ (6 October 2020) <https://level.medium.com/the-demand-for-abolition-979c759ff6f>.

168
*Callins v Collins* 510 US 1141 (1994).

169 See Bernstein, ‘Carceral Politics’ (n 165); Tapia Tapia, ‘Feminism and Penal Expansion’ (n 10) 292.

170 See L Fekete, ‘Europe: Crimes of Solidarity’ (2009) 50 Race & Class 83; United Nations Special Rapporteur on Extrajudicial, Summary or Arbitrary Executions, ‘Thematic Report: Saving Lives is Not a Crime’ (6 August 2018) UN Doc A/73/314.

171 See eg EA Stanley, D Spade and Queer (In)Justice, ‘Queering Prison Abolition, Now?’ (2012) 64(1) American Quarterly 115; A Aliverti and others, ‘Decolonizing the Criminal Question’ (2021) 23(3) Punishment & Society 297.

172 K-Y Taylor, ‘The Emerging Movement for Police and Prison Abolition’ *The New Yorker* (7 May 2021).

173 See eg B Pali and V Canning, ‘Challenging Co-optive Criminalisation: Feminist-Centred Decarceration Strategies for Interpersonal and Sexualised Violence’ (2022) 61(1) The Howard Journal of Crime and Justice 68; A Chamberlen and H Carvalho, ‘Feeling the Absence of Justice: Notes on Our Pathological Reliance on Punitive Justice’ (2022) 61(1) The Howard Journal of Crime and Justice 87; S Aiken and S J Silverman, ‘Decarceral Futures: Bridging Immigration and Prison Justice towards an Abolitionist Future’ (2021) 25(2) Citizenship Studies 141.

174 On this potential within human rights, see K McNeilly, *Human Rights and Radical Social Transformation: Futurity, Alterity, Power* (Routledge 2019). But see Kapur (n 7).

175 In this respect, see the critique of individual criminal accountability as obscuring the responsibility of states, in LE Fletcher, ‘A Wolf in Sheep’s Clothing: Transitional Justice and the Effacement of State Accountability for International Crimes’ (2015) 39 FILJ 447.

176 See further M Pinto and N Mavronicola, ‘Accountability for Violations of Civil and Political Rights: Redress Within and Beyond the Penal Frame’ (on file with author).

177 On the second-class status of socio-economic rights, see D Barak-Erez and A Gross, ‘Introduction: Do We Need Social Rights?’ in D Barak-Erez and A Gross (eds), *Exploring Social Rights* (Hart Publishing 2007) 6. On the importance of socio-economic rights and obligations in the aftermath of serious violence, see F Torres, ‘Reparations: To What End? Developing the State’s Positive Duties to Address Socio-economic Harms in Post-conflict Settings through the European Court of Human Rights’ (2021) 32(3) EJIL 807; F Torres, ‘The State, the Assailant? Guaranteeing Economic and Social Rights after Widespread Violence through the Inter-American Court of Human Rights’ (2022) 40(1) NQHR 12.

## Notes

This article is current as at 31 August 2023. The article benefited enormously from a Modern Law Review-funded seminar on ‘Human Rights Penality’ in March 2022, and multiple exchanges with a range of wonderful scholars, including: Julia Anderson, Aristotelis Constantinides, Fiona de Londras, Karen Engle, Damian Gonzalez-Salzberg, Zehrah Hasan, Corina Heri, Laurens Lavrysen, Steven Malby, Mattia Pinto, Adnan Sattar, Silvana Tapia Tapia, Dirk Van Zyl Smit, Irene Wieczorek, and colleagues attending my talks at the University of Cambridge, University of Cyprus, University of Durham, University of Leicester, University of Liverpool, Lund University and iCourts (University of Copenhagen), as well as my students at Birmingham Law School. I am grateful to the anonymous referees for their invaluable engagement with and feedback on the piece. Any errors or oversights remain my own. The author is grateful to the Modern Law Review for funding a workshop in which this paper was elaborated and reviewed.

